# Impact of co-created mental health and life-skill workshops with 12-to-16-year-olds from black and mixed ethnic groups during COVID-19 in the UK: a qualitative study

**DOI:** 10.1136/bmjopen-2025-107310

**Published:** 2026-05-26

**Authors:** Isabelle Pomfret, Keri Ka-Yee Wong

**Affiliations:** 1 GOS ICH, University College London, London, UK; 2 Department of Psychology and Human Development, Institute of Education, University College London, London, UK; 3 UCL Centre for Education and Criminal Justice, London, UK

**Keywords:** MENTAL HEALTH, Psychosocial Intervention, QUALITATIVE RESEARCH

## Abstract

**Abstract:**

**Background:**

The COVID-19 pandemic has disproportionately impacted the mental health of young people from minority ethnic communities, yet effective interventions such as mental health psychoeducational workshops, shown to work for other populations, have rarely been offered or investigated among this population.

**Objectives:**

This qualitative study examines the impact, challenges and benefits of mental health psychoeducational workshops co-created with and for 12-to-16-year-olds from black and mixed ethnic minority groups in London, UK.

**Methods:**

12 (8 female: 3 male) black and mixed ethnic minority 12-to-16-year-olds (*M*=16, SD=1.55 years) currently attending a West London community centre co-created, participated in, and fed back on the impact of five mental health and life-skill workshops through one-on-one semi-structured interviews (*M*=10 min 8 s, range=3–16 min), which were transcribed verbatim.

**Findings:**

Interpretative Phenomenological Analysis of the interviews revealed three superordinate themes, with a total of eight codes clustered: (1) workshop features promoting positive mental health, (2) positive mental health outcomes and (3) workshop features impeding positive mental health outcomes. Overall, young people perceived workshops to have a positive impact on their mental health and helped provide support in coping with the challenges of the COVID-19 pandemic.

**Conclusions:**

Study findings highlight the potential benefits and barriers to entry of mental health and life-skill workshops for young people from minority ethnic communities.

**Clinical applications:**

Community-based and co-produced workshops were perceived as beneficial to mental health by Black, Asian, Minority Ethnic young people, warranting greater consideration and implementation in practice.

**STRENGTHS AND LIMITATIONS OF THIS STUDY**

- One-to-one in-depth interviews provided rich data for Interpretative Phenomenological Analysis, through which young people’s lived experiences and perceptions were uncovered.
- Participatory approach with young people throughout the project timeline enhances contextual relevance and participant engagement in the research process.
- The semi-structured interview guide allowed for consistency across interviews while permitting flexibility, enabling individual experiences and opinions to shine through.
- An iterative and thorough coding process allowed themes to be developed naturally and inductively from the data.
- The small sample size (n=12) of participants from a wide age range (12–16) from a single West London community centre limits the generalisability of findings to other settings.

## Background

 2020 saw the spread of the severe acute respiratory syndrome coronavirus COVID-19 across the world, which infected 764 million people and killed 7 million worldwide.^1^ Strict lockdown measures, though necessary to contain the spread of the disease, caused significant economic, social and psychological consequences for all and blurred our relationships with the spaces in which we learn, work and live.^2 3^ Global prevalence of anxiety and depression increased by 25% with studies documenting the ongoing consequences of anxiety, social isolation, loneliness, economic uncertainty and health and well-being concerns for the vulnerable.^4 5^ 4 years and counting since lockdowns and the peak of the pandemic, negative mental health continues to impact the general population, particularly for those living with existing vulnerabilities.^3^

One group that is particularly affected are adults and children from minority ethnic communities. Studies have shown that Black, Asian, Minority Ethnic (BAME) groups were found to be more susceptible to experiencing financial instability, unemployment and food insecurity^6^,^8^ and reported higher levels of psychological symptoms such as depression during the COVID-19 pandemic.^6^ Data from the UK and globally suggest that BAME groups were at greater risk of dying from COVID-19. For example, the standardised mortality ratio (SMR) for White British was 0.88 (95% CI 0.86 to 0.89), much lower than the 3.24 SMR (95% CI 2.90 to 3.62) for Black Africans.^9^ For the latter group, young people from BAME communities were especially vulnerable, as evidenced by sharp increases in helpline requests from BAME under-18’s than White peers of the same age group (based on 51 321 total requests during 2020).^10^ BAME young people reported increased levels of anxiety (11.4%), suicidal thoughts (26.6%) and self-harm (29.5%), compared with their White peers at 3%, 18.1% and 24.9%, respectively.^10^ Uneven mental health access intensifies this issue.^11^ BAME groups are less likely to receive necessary mental healthcare and more likely to delay seeking help due to stigma around mental health.^12^ ^11 13^ Institutional injustice was widely reported, with BAME people feeling disregarded and underserved by mental healthcare institutions in the UK.^13^ Taken together, young people from BAME communities should be prioritised for health support.

A key challenge is that mental health concerns of BAME young people are rarely discussed, and hence, pathways to support them remain unclear. To date, only a handful of studies have focused on understanding how young people from BAME communities struggled with heightened socioeconomic and psychological challenges during the COVID-19 pandemic (Lakhanpaul *et al*,^14^ Burgess *et al*^15^ and Lee and Wong.^16^ In a separate study, Lenoir and Wong^17^ also identified the mental health struggles and the important areas of support needed by BAME young people during the COVID-19 pandemic, including addressing low self-esteem, lack of motivation and isolation, though there were also positive outcomes too (positive solitude and new connections). Other documented issues include poor communication, lack of trust in research and the hidden costs of research participation.^18^ ^19^ More research is needed to uncover the voices of young people from BAME communities, particularly through co-design research principles.^19^

While support in clinical settings is available, they are less accessible to young people from BAME communities. However, alternative community-based psychoeducational workshops delivered through youth clubs and/or local councils have shown promise. Baskin *et al*^20^ conducted a scoping review of the effectiveness of such community-centred programmes for ethnic minority adults in the UK, finding reductions in social isolation and stress and improvements in mood and self-confidence. Conversely, Slattery *et al*’s qualitative longitudinal study^21^ found that socialisation between participants in a group during creative and psycho-social workshops facilitated mental health recovery, even though study participants were adults from non-minority ethnic groups. This social element may be especially important since many individuals had limited socialisation and felt isolated/lonely during the COVID-19 pandemic.^5^ Co-created workshops were also tested in Dunn’s study,^22^ where young people highlighted the importance of support workers in facilitating the programme’s success. Although there are clear advantages of co-created workshops with and for young people, few have assessed which aspects and how workshops may be effective for BAME young people’s health and development, particularly during a time of global stress and pandemic.

The current semi-structured interview study, part of the larger UKRI Research England-funded CopeWell study^23^ explores the impact of a series of mental health and life-skill workshops co-created with and for young people from BAME communities who regularly attend a West-London youth club. The study’s aim is twofold: (i) to explore BAME young people’s perception of the role of co-created mental health awareness workshops in promoting positive mental health during COVID-19 and (ii) to identify which aspects of the co-created workshops were most and least beneficial to inform future community-based programmes.

## Objective

To examine the impact, challenges and benefits of a series of co-created mental health psychoeducational workshops as voiced by 12-to-16-year-olds from Black and Asian mixed ethnic groups.

## Methods

### Participants

12 adolescents aged 12–16 years (*M*=16 years, SD=1.92 years; female=72.3%) took part in the study, with one participant not disclosing age or ethnicity data. Participants self-reported as identifying with the following ethnic groups: Black or Black-African (n=4), Mixed-White and Black African (n=2), Mixed-White and Black Caribbean (n=1), Black or Black-Caribbean (n=1), any other Black background (n=1) and preferred not to say (n=2). Participants were recruited through convenience sample from a West London youth community centre. The full participant characteristic table is included in online supplemental appendix A.

Participants took part in at least one of the five in-person workshops in the CopeWell Study between 26 March 2022 and 31 May 2022. Workshops were co-created and psychosocial in nature, incorporating mental health themes, therapeutic elements and practical skills as part of the pre-registered CopeWell study. These topics were identified independently through one-on-one interviews and focus groups during the study’s inception phase (see the OSF page https://osf.io/jcak7/ and study report for workshop details). A total of five in-person workshops were co-created and delivered with and for participants directly to address the impact of the COVID-19 pandemic on their lives. Workshop 1 involved participants taking a campus tour to introduce them to the university environment. Workshop 2 involved a journalist who spoke about the power of stories and the interpretation of our own story and experiences before sharing their career journey. Workshop 3 focused on body image and the impact of social media, featuring a talk by a clinical psychologist on coping skills. Workshop 4 involved social psychologists who led conversations on in-group/out-group identity, exploring topics such as discrimination, prejudice and stereotypes. Workshop 5 concluded with a creative session in which participants and members of the research team co-produced a single piece of artwork reflecting on what they had learnt throughout the programme. At the end of the roughly 3 month series of workshops, participants provided one-on-one interview feedback to three undergraduate co-researchers on 6 July 2022 at the youth centre. Workshop attendance ranged from 8 to 15 participants, with around 20 young people regularly attending this youth club. This interview audio was recorded and transcribed verbatim by two third-year undergraduate student co-researchers (IP and ZBD). Interviews ranged from 3 to 15 min (*M*=8.21 min) and no field notes were taken. On completing the feedback, participants were debriefed and received a £30 gift voucher of their choice. Informed parental consent was sought beforehand, and participants signed a written consent at the time of the interviews. Participants were reminded of their right to withdraw from the study at any time, given the opportunity to ask questions, and informed how their data would be used. No participants dropped out of the study partway. The same group of student researchers were consistently present at the youth centre, in workshop sessions and in conducting the focus groups and interviews, strengthening their ties with the young participants with each interaction.

## Materials

A semi-structured interview schedule consisting of 10 open-ended questions designed by KK-YW and administered by student co-researchers (see online supplemental appendix B). Questions were made to assess the young person’s enjoyment of the workshops, the workshop’s impact on their mental health and which aspects of the workshop were and were not beneficial.

### Data analysis

Audio files were transcribed verbatim, and transcripts were compared between two transcribers to corroborate the reliability and accuracy of the full transcript. IP completed a checklist for qualitative research Consolidated criteria for Reporting Qualitative research (COREQ) (see online supplemental appendix C). Data were analysed using Interpretative Phenomenological Analysis (IPA), a commonly used method to capture subjective experience.^24 25^ IP conducted IPA using Smith and Shinebourne’s^25^ bottom-up six-step process and referring closely to Nizza *et al*’s^26^ criteria for achieving excellence in IPA. IP engaged in a close analytical reading of the transcript, highlighting and exploring quotes that informed emergent themes. Patterns were established by identifying connections among these themes. This process resulted in *superordinate themes*, *themes* and *subthemes* presented in descending order of superiority.

Relevant quotes to the study aim were synthesised using NVIVO V.12. Each quote was scrutinised carefully, and its significance explored both individually and in the context of the wider transcript.^26^ By comparing the similarities and differences between participants, IP also established patterns between top-level qualities, simultaneously highlighting the uniqueness of each account.^26^ Recurrent pattern clusters informed the generation of themes and subthemes (online supplemental appendices C and D), and each theme was constructed and arranged to add to a larger narrative of the significance of workshop experiences.^25^ Through refinement and comparison, themes were grouped into three superordinate themes. We achieve saturation through an iterative process where IP read and reread the transcripts, updated the coding book, and when no new codes or thematic insights were generated, and all subsequent data could be fully accommodated within the existing coding framework (see online supplemental appendix D—Final Thematic Tables). IP worked with a second researcher (ZBD) to cross-validate the themes and achieve inter-rater reliability during coding, throughout which KW also reviewed the codes and gave feedback on the iterative process. For completeness, a second researcher trained in the codes cross-checked the final transcript and agreed with the choice of themes, a crucial step in a subjective process like IPA.^25^ However, transcripts and ultimate findings were not returned to participants for comment or correction.

### Patient and public involvement

Young people who regularly attended a West London community centre youth club took part in interviews and focus groups that informed the creation of the workshop, as reported in Lenoir and Wong.^16^

## Findings

Three superordinate themes and eight themes were identified through IPA analysis. Themes, subthemes and illustrative quotes are described in tables1^3^.

**Table 1:** Aspects of the workshops promoting positive mental health outcomes

Themes	Subthemes	Illustrative quotes
Enjoyment and engagement	Enjoyment of the workshops	P12: “I think I enjoyed the BBC one a lot because I resonated with it very closely due to the fact, I love filmmaking and directing and movies and acting and whatnot” P7: “But it was really fun. That part of it and it was kinda interactive, so it wasn’t really that boring.”
	Engagement with the workshop activities	P7: “I found it quite engaging um because yeah I was like actually interested about it.”
Community building	Social interaction	P10: “Doing it with your friends is a lot more fun as well.” P6: “It was good like to know people cause like now I know you”
	Social empathy and understanding	P11: “Well it helped me learn about people’s insecurities and why they have them and also it helped me as well to understand other people.” P8: And, it was really, it’s nice to hear other people’s point of views and perspectives so I kind of enjoyed the sit-down talk and everyone was just saying their opinions.”
Coping strategies		P3: “Well I think the mental health one helped like the people understand like what to do if you’re like um like if you can’t concentrate or if you’re not calm like you can go on walks and stuff. It helped us think about what you can do.” P12: “It helped, they gave us some like exercises and things that we can do and I feel like every time I feel like really anxious or frustrated, I think back to that and then and then I’m like OK take a couple of deep breaths and yeah it like helps me feel better.”

BBC, British Broadcasting Channel.

**Table 2:** Positive mental health outcomes

Themes	Subthemes	Illustrative quotes
Self-understanding	Self-reflection	P8: “you kinda learn things about yourself in a way” P12: “Yeah the body image yeah it was a lot about body confidence, and I think I was going through something at that time with my body image and I think that workshop was like a godsend like it really opened my eyes a lot”
	Change in negative mental health perception	P4: “so sometimes you’ll dwell on something which isn’t actually isn’t as a big as it is and you think if a random stranger was to see you on the street was it really the first thing they’d notice?” P5: “Um I mean I learned that uh people stereotyping isn’t like good. It’s like- even jokes like you can’t really make jokes of it cause it’s not funny at the end cause like it could be affecting somebody’s life.”
Emotional expression and regulation	Emotional expression	P9: “I would say, I would say like it’s life changing to the point like you have to express you have to talk like some people out here do not talk to nobody” P10: “Not really career paths but it changed my perception on the social identity and stereotypes”
	Emotional regulation	P8: “It was quite calming to be honest” P12: “So I feel like these workshops helped me a lot with my emotions.”

**Table 3:** Aspects of the workshop which impeded positive mental health outcomes

Themes	Illustrative quotes
Negative perception	P2: “To be honest with you I didn’t really- I just thought it was going to be like a boring day at school” P12: “Because I feel like most people when they see it for the first time they’re bored or they’re only in it for like the vouchers”
Lack of engagement	P7: “No offence to the people doing the BBC one but it was absolutely boring. I left halfway through and then I had to come back cause I was forced back but like it was really boring and they were telling like all about themselves and I know it was rude to leave but I was so bored don’t blame me.” P9: “Like, I don’t know. I like art but it wasn’t like, it wasn’t, it was just dry.”
Topic irrelevance	P6: “My mum calls- like my mum would call me fine and then my sister would call me like skinny but then they all would call me fat so like for them I’m everything. For me I just don’t really care. Like who cares?” P7: “Well, I don’t think about my mental health. I actually try and stay as far away from it as possible.”

BBC, British Broadcasting Channel.

### Superordinate theme 1: workshop features promoting positive mental health outcomes

#### Enjoyment and engagement

Nearly all participants (n=10) noted that the workshops were enjoyable in some way and described meaningful engagement with activities that aligned with their interests. Participants suggested that this engagement positively affected their mood, which in turn supported their overall involvement in sessions.

I enjoyed the BBC one a lot because I resonated with it very closely due to the fact I love filmmaking and directing (ID103, aged 16, female)

#### Community building

A recurring theme was the positive nature of the social relationships formed through mutual engagement in workshops. The majority of participants (n=9) described how they enjoyed getting ‘to know people’ and that this collaboration made one ‘feel part of a group’. Adolescents also reported developing a greater understanding or empathy for others’ mental health. The lingering shadow of the pandemic appeared to shape these responses, as participants expressed a particular appreciation for this face-to-face opportunity to engage with peers of a similar age group.

It helped me learn about people’s insecurities and why they have them and also it helped me as well to understand other people. (ID112, aged 15, male)

#### Coping strategies

Coping strategies and skills taught in the workshops, including mindfulness, art and relaxation techniques, were found helpful by some (n=3) in managing negative emotions such as anxiety and frustration. Teaching these strategies in a practical and accessible way may enable participants to apply them beyond the workshop setting.

It helped, they gave us some like exercises and things that we can do, and I feel like every time I feel like really anxious or frustrated, I think back to that and then and then I’m like OK, take a couple of deep breaths. (ID103, aged 16, female)

### Superordinate theme 2: positive mental health outcomes

#### Self-understanding

Many of the participants (n=8) spoke about the workshops as an opportunity to look inward and reflect on their mental health journeys. Through greater understanding of mental health topics, they ultimately recognised feelings that they were not previously aware of or had not addressed. Workshops were a safe space among a group of understanding peers where participants could truly reflect on the lingering mental health toll of the pandemic, some for the first time.

It helps me a lot with realising that like I’m not perfect…All I can do is trying to be the best I can be, but I know that’s not going to be perfect and that doesn’t have to be perfect for me to be good enough*.* (ID103, aged 16, female)

#### Emotional expression and regulation

For several participants (n=4), the workshops helped them articulate both positive and negative emotions through verbal and creative means. Often, these emotions were related to lingering anxieties that had emerged during the pandemic. These workshops emphasised the importance of acknowledging and processing negative emotions, rather than suppressing them, as a fundamental step toward emotional well-being.

I would say, I would say like it’s life changing to the point like you have to express you have to talk like some people out here do not talk to nobody (ID102, aged 16, female)

### Superordinate theme 3: workshop features that impeded positive mental health outcomes

#### Negative perceptions

Several participants (n=3) noted that they initially held a negative perception of the workshops, assuming that they would be boring or not worthwhile. Although these views were challenged over the course of the workshops, such negative preconceptions may nevertheless act as barriers to receiving mental health and educational benefits, particularly for young people from BAME backgrounds. Another concern raised was that some individuals were ‘only in it for like the vouchers’, raising the issue of monetary incentives artificially inflating attendance and engagement.

#### Lack of engagement

There was a tendency for some participants (n=4) to lose engagement when the session activities did not resonate with them, either electing to leave altogether or losing focus. This may be due to participants’ age, as those who expressed this lack of interest were among the youngest in the group. For example, one 12-year-old male participant noted that ‘the BBC one (workshop)…was absolutely boring. I left halfway through*’.*

#### Topic irrelevance

Some of the topics chosen for the workshops were perceived as irrelevant, uninteresting or too niche. A few participants (n=2) also did not have a previous mental health issue related to the topics of the workshop and therefore did not feel represented.

I feel like that’s a very, I feel like with the art one (workshop) there’s only certain people that like art, with BBC there’s only certain people that like directing*.* (ID103, aged 16, female)

## Discussion

The current study examined the role and pros and cons of co-created mental health workshops in promoting positive health in young people from black and mixed-ethnic communities during COVID-19. Crucially, the workshops were delivered in person shortly following the easing of COVID-19 restrictions, and the sessions represented a meaningful opportunity to re-engage in face-to-face group activities. Overall, participants found the workshops generally positive for their mental health, though there were some suggestions for improvement in future workshops. Each of these findings is discussed in turn.

Participants described the workshops as ‘very entertaining’ but also ‘eye-opening’ and ‘a good escape’ from the ‘pandemic’ (Superordinate theme 1). They also spoke about positive social interactions with others during the workshops, ‘making new connections with other people’ and ‘[getting] to hear other people’s points of view and perspectives’. Prior research has demonstrated that social connection is an essential thematic element of workshops with links to positive outcomes,^15^ and this is perhaps unsurprising given the importance of social relationships and the influence of peers in young people’s lives during middle childhood.^27^ New friendships not only enhanced the enjoyment of the workshop but also promoted social skills and normalised sharing mental health struggles with others (eg, ‘more confident to talk to other people, explain how [they] feel’). In the context of the pandemic, the workshops unquestionably helped fill a pressing gap in face-to-face social interaction. Moreover, the workshops stood out against social activities as a uniquely structured space where the expression of vulnerable emotions and meaningful personal experiences was explicitly encouraged. Participants may have felt an even deeper connection to their peers as a result of these honest conversations.

Participants also spoke about the coping strategies learnt and the actionable steps to take when they felt mentally overwhelmed. This ‘helped [them] a lot because [they] never really had like a sort of system… to deal with the negativity’ but that ‘everytime [they] feel really anxious or frustrated, [they] think back to that (the coping exercises) and then… it helps [them] feel better.’ This suggests that some learning has been retained by the participants post-workshop, as the feedback took place at the end of a year-long project. Similar benefits are echoed in Sharifi *et al*’s study,^28^ in which participants found learning coping strategies, such as relaxation techniques, helpful for managing their own emotional distress and supporting those around them. However, whether even longer-term workshop impacts are realised will require a future follow-up. It is regrettable that since the pandemic was sudden and unexpected, adolescents may not have had the time beforehand to develop or practise healthy coping mechanisms. With the benefit of hindsight, workshops can now equip participants with the tools to rebuild crucial emotional regulation skills and carry them through future periods of instability.

Superordinate theme 2 described the ‘positive mental health outcomes from workshop participation’. One young person felt that the workshops, specifically the body image workshop, ‘helped them a lot with understanding [their] emotions and connecting with them’, and positive messaging helped them empathise with and accept themselves as they are, realising that ‘they were a very emotional person… [but that] it’s okay to be emotional’. One participant described how timely the workshop felt in relation to their own struggles, explaining, “I think I was going through something at that time with my body image and I think that workshop was like a godsend.” Self-acceptance was a common theme, with another adolescent also ‘realis[ing] that… [they were] not perfect’ but that they ‘[didn’t] have to be perfect… to be good enough’. These findings mirror those from Ahorsu *et al*’s study^29^ where psychosocial information helped workshop participants better evaluate their own mental distress and feel more in tune with their emotions. The workshops appeared to provide not only information but also emotional validation, enabling participants to reframe negative self-perceptions and feel less alone in their experiences. For some participants, this process of reflection and discussion facilitated a shift towards greater self-compassion and confidence.

Against the backdrop of the workshop benefits, theme 3 identifies several areas for improvement. Some participants said that different workshop sessions did not appeal to them, such as the BBC (British Broadcasting Channel) workshop being ‘absolutely boring’ or the art workshop being ‘dry’. This translated into the lack of engagement during workshops, with avoidance behaviours entirely (eg, ‘walked out’). Others initially had a negative perception of workshops but later changed their minds and found the workshops helpful. Negative initial perceptions can affect sign-ups, sustained interest and commitment to programmes when young people are unaware of the workshop’s benefits. Negative perceptions of mental health, distrust in research, negative experiences of discrimination or a lack of prioritisation^13^ may already exist among BAME communities and should be acknowledged in future workshop design. In Dunn’s study, young people viewed already known and trusted peer transition support workers as a key beneficial element of the programme, emphasising the need for closer partnership with community youth and social workers.^22^ While workshops may not cater for everyone’s needs in the community, it is important to recognise that the wide age range in participants did add to the challenge. In particular, younger participants found the sessions boring, despite the sessions being co-created by the same group of young people. In addition to future workshops being youth-led, relevant and interesting, the age group they are offered to should also be considered. Another interesting point raised was that providing vouchers may have unintentionally emphasised financial incentives over authentic interest in the workshop content, particularly for a group living with existing inequalities. This issue is complex and has been widely debated previously, yet it is not a key focus of the current study.^30^

### Strengths and limitations

A key strength of this study was its IPA analysis, an idiographic method that allows the experiences of underrepresented groups to be authentically expressed and contextualised in their own terms.^25^ Semi-structured interviews allowed participants to expand on their thoughts or lead the interviews in new, unanticipated directions. A second strength was that participants could relate to student co-researchers of similar age and developed strong relationships, which may have contributed to more honest feedback by the end. A third strength, interviews were conducted only 1 week after the fifth and final workshop was complete, minimising recall bias

However, this study is not without limitations. Participants may also have felt more compelled to provide positive workshop feedback during a non-anonymous interview with an actively involved researcher. Another limitation is the wide age range of participants (12–16 years), which is reflected in the variety of workshop experiences, but also highlights potential areas for improving future workshop specificity. Due to time restraints, participants also did not provide feedback on the study findings, so it is unknown whether they intended different meanings from the interviews. The sample size was small, with convenience sampling from a single community centre. However, the experiences captured in this study nonetheless represent views from an underrepresented population and their views are valuable in informing support.^19^

Findings can inform existing community-based initiatives by including co-produced activities tailored to a narrower age range, as well as youth provision by local authority social workers, practitioners and teachers. Additional support for existing grassroots charities working with young people from ethnic minority backgrounds should be prioritised. These insights can inform future low-cost, community-based, co-created workshops in youth clubs and community centres and directly address government policies on high-impact preventive healthcare interventions. Future research should expand the scope of this study to include a more ethnically diverse sample and a control group to better understand culturally specific needs. The further impact of creative workshops embedded in schools and after-school activities may warrant further investigation.

## Conclusion

This semi-structured interview study investigated young people from minority ethnic communities’ experiences of co-created mental health and life-skill workshops and their impact as they continue to recover from the COVID-19 pandemic. The themes highlighted how the workshops imparted crucial well-being skills, such as emotional regulation, self-acceptance and long-term coping strategies, alongside social benefits, fostering a truly supportive community among their participants. Collectively, these elements supported participants’ capacity to navigate ongoing challenges during post-pandemic recovery. Future workshops should prioritise participant enjoyment, engagement, community building and the coping skills gained from attending, and continue to tailor community support for young people. This workshop feedback is but a start to a critical area of strategic community preventative intervention for mental well-being.

## Supplementary material

10.1136/bmjopen-2025-107310 online supplemental file 1

## Data Availability

Data are available in a public, open access repository.
