# Supplementary material for: Impact of co-created mental health and life-skill workshops with 12-to-16-year-olds from black and mixed ethnic groups during COVID-19 in the UK: a qualitative study
Source: BMJ Open. 2026 May 26;16(5):e107310. doi: 10.1136/bmjopen-2025-107310 (PMC13218165; doi:10.1136/bmjopen-2025-107310)
Supplement: online supplemental file 1 [file bmjopen-16-5-s001.pdf]

Appendix A

Participant Characteristics Table

Table A1. *Participant Characteristics Table*

Demographics	Description	Number
**Age**	12	1
	13	2
	14	1
	15	6
	16	1
**Gender**	Male	7
	Female	12
**Ethnicity**	Black or Black-African	4
	Any other Black background	1
	Mixed – White and Black African	2
	Mixed – White and Black Caribbean	1
	Black or Black-Caribbean	1
	Prefer not to say	2

Appendix B

Final Interview Schedule

1-on-1 session (RA Instruction):

"In this session we're wanting to learn from you to see how we can improve on the workshops in the future. Here are all the workshops that took place. Have a look through, pick the ones that most memorable for you."

Q: Which workshop(s) did you enjoy the most and why?

Q: Are there specific **activities, conversations, speakers, or places** which was most memorable to you and why?

Q: Across the workshops, how has taking part **helped you** in any way?

Q: Have you developed any **new skills** from taking part in the study?

Q: How have these workshops shaped **how you think about yourself and others**?

Q: How have these workshops shaped **how you think about mental health**?

Q: How have these workshops shaped **how you think about university**?

Q: How have these workshops shaped **how you think about the covid pandemic**?

Q: If you were to tell other young people about the CopeWell Study workshops, what would you say?

Q: Would you recommend these workshops to other young people your age? If so, which workshops? If not, why not?

Record Soundbite: Pretend you are now telling another young person who has not taken part in the CopeWell Study workshops before - what message would you say to them?

Record Soundbite: If you could tell the people who funded these workshops about your experience, what would you say?

Appendix C

COREQ Checklist

COREQ (COnsolidated criteria for REporting Qualitative research) Checklist

A checklist of items that should be included in reports of qualitative research. You must report the page number in your manuscript where you consider each of the items listed in this checklist. If you have not included this information, either revise your manuscript accordingly before submitting or note N/A.

Topic	Item No.	Guide Questions/Description	Reported on Page No.
**Domain 1: Research team and reflexivity**			
Personal characteristics			
Interviewer/facilitator	1	Which author/s conducted the interview or focus group?	7
Credentials	2	What were the researcher's credentials? E.g. PhD, MD	7
Occupation	3	What was their occupation at the time of the study?	7
Gender	4	Was the researcher male or female?	7
Experience and training	5	What experience or training did the researcher have?	7
Relationship with participants			
Relationship established	6	Was a relationship established prior to study commencement?	7
Participant knowledge of the interviewer	7	What did the participants know about the researcher? e.g. personal goals, reasons for doing the research	7
Interviewer characteristics	8	What characteristics were reported about the inter viewer/facilitator? e.g. Bias, assumptions, reasons and interests in the research topic	7
**Domain 2: Study design**			
Theoretical framework			
Methodological orientation and Theory	9	What methodological orientation was stated to underpin the study? e.g. grounded theory, discourse analysis, ethnography, phenomenology, content analysis	8
Participant selection			
Sampling	10	How were participants selected? e.g. purposive, convenience, consecutive, snowball	7
Method of approach	11	How were participants approached? e.g. face-to-face, telephone, mail, email	7
Sample size	12	How many participants were in the study?	6
Non-participation	13	How many people refused to participate or dropped out? Reasons?	7
Setting			
Setting of data collection	14	Where was the data collected? e.g. home, clinic, workplace	7
Presence of non-participants	15	Was anyone else present besides the participants and researchers?	7
Description of sample	16	What are the important characteristics of the sample? e.g. demographic data, date	7
Data collection			
Interview guide	17	Were questions, prompts, guides provided by the authors? Was it pilot tested?	7
Repeat interviews	18	Were repeat inter views carried out? If yes, how many?	7
Audio/visual recording	19	Did the research use audio or visual recording to collect the data?	7
Field notes	20	Were field notes made during and/or after the inter view or focus group?	7
Duration	21	What was the duration of the inter views or focus group?	7
Data saturation	22	Was data saturation discussed?	9
Transcripts returned	23	Were transcripts returned to participants for comment and/or	9

Appendix D

Final Thematic Tables

Table D1.

All quotations related to superordinate theme 1: aspects of the workshops promoting positive mental health outcomes

Superordinate theme 1: Enabling factors for positive mental health outcomes		
Enjoyment and engagement	Community building	Coping strategies
Enjoyment<br>P2: "like you were learning things as well and you were having fun"<br>P3: "I liked- my favourite one was the BBC and the body image cause they both included lots of things"<br>P5: "Yeah, and my experience was good uh I liked it"<br>P5: "Yeah, I'd recommend it. It was fun, it was good."<br>P5: "I enjoyed it."<br>P5: "it really helped to like experience like the real world like you don't know what's happening so like if you go there you can find out new things"<br>P6: "because like I enjoyed it like coming to uni not coming to us like-<br>P7: "It was still really fun."<br>P7: "The first workshop was the most fun."<br>P7: "But it was really fun. That part of it and it was kinda interactive so it wasn't really that boring. That kinda sounds rude doesn't it."<br>P7: "Would you recommend…? One, yes. Because it's fun and you go to lots of different places."<br>P7: "But it was still fun. Erm, and I would recommend it to you whoever you are."<br>P8: "I learned more about little things that I found enjoyment in"	*Social connection*<br>P2: "you were making new connections with other people as well so yeah."<br>P2: "they included everyone to speak"<br>P6: "it was good like to know people cause like now I know you"<br>P7: "Well, I kinda got to know everyone a little bit more and stuff that I didn't know about them"<br>P8: "At that time, we were all sitting down in a circle, and everyone did say a question, and everyone would say their personal views and opinions on it, and it would lead to some type of debate. It's like there was always a question for a question and everyone would say their own input. And, it was really, it's nice to hear other people's point of views and perspectives so I kind of enjoyed the sit-down talk and everyone was just saying their opinions."<br>P8: "learned a lot about it in teamwork as well."<br>P8:" but hearing everyone else's experiences because that what we talked about first, that was pretty like, everyone had very much I thought we all but, everyone had very different experiences towards the pandemic so hearing about	P3: "Well I think the mental health one helped like the people understand like what to do if you're like um like if you can't concentrate or if you're not calm like you can go on walks and stuff. It helped us think about what you can do."<br>P3: "Try to like get them out of the situation and make sure they don't you know come in contact with that."<br>P12: "sometimes it's not about like what it does for the like what it does physically it's more about what it can do for you mentally like in the future and what not it's not really, it's not always about what's happening right now. It's about what could happen if you like paid attention and listened and what not."<br>P12: "Yeah, I feel like one of the workshops it was talking about how to combat negative emotions. I feel like that helped me a lot because I never really had like a sort of system or like coping mechanisms to deal with the negativity. I just sort of pushed it down and let it all bottle up and then I explode and then I realised that that's not healthy for me and yeah. It helped, they gave us some like exercises and things that we can do

P8: “but I actually enjoyed it.”
P8: “I kind of liked the art. The art was really nice”
P8: “I had a pretty good experience I would say. Like there was nothing really wrong, were nice people, they showed us around campus, they provided food, they provided activities, talks. Erm we had laughs there was like- it was pretty much like having a regular day.”
P9: “Yeah like it was, I loved it.”
P9: “I kind of forgot about the pandemic like, yeah, I don’t like, the workshops made me forget. That’s how fun it was.”
P10: I’m a chef myself. I do Italian- I do Italian cuisine so I like eating food and it was Italian food, so I like eating it even more!
P10: “I got to share some fun facts which was, you know, fun”
P10: “but it was very entertaining.”
P10: “It was mostly fun”
P12: “I think I enjoyed the BBC one a lot because I resonated with it very closely due to the fact I love filmmaking and directing and movies“
P12: “I realised it was quite fun because I got to understand what they did”
P12: “yeah it was just a very like eye opening experience and I got to contribute a lot to it because that’s like something I like a lot like I love BBC.”
P12: “Because I feel like it’s a good escape”

Interest and engagement
P1: “It did that’s why I put my hand up at one point and I said aw like there’s some it after the whole pandemic and talking to everybody, it was pretty nice.”
P9: “I would say, I would say like it’s life changing to the point like you have to express you have to talk like some people out here do not talk to nobody and with these workshops it makes you more confident to talk to other people, explain how you feel, yeah.”
P9: “you get to speak to other people that you don’t know.”
P10: “Doing it with your friends is a lot more fun as well.”
P11: “Um well about talking about mental health um well I enjoy like talking about it with other people”
P11: “Well I enjoy working with them because it makes me feel like part of their group. Also, the other people as well I kept talking with them as well and like specialists.”
P12: “like it’s a good way to connect”

Social empathy and understanding
P1: “like it makes us see other people differently type thing and yeah.”
P1: “Um I would say like especially to the girls out there that do feel insecure about themselves I would like I’ll tell them to go to the workshops like specifically and tell them like you shouldn’t worry about you’re thinking like other people will tell you yeah you look- like you’re perfect shouldn’t be insecure about yourself”
P6: “Because I feel like my friends are always talking about their bodies.”
P6: “It might change how they feel about themselves” and I feel like every time I feel like really anxious or frustrated, I think back to that and then and then I’m like OK take a couple of deep breaths and yeah it like helps me feel better.”

points where I'm like yeah like I am pretty girl but when I look at instagram and all of that then then I'm like aw but maybe I'm not so pretty like you get it so?"<br>P2: "when she was kinda like speaking to us about it at first I was kinda having like doubts about uni but then I don't know like when she spoke to me it's like mm maybe there's actually kind of hope"<br>P3: "I learned about other people's jobs and although it's just one job like the BBC its news reporting it comes with loads of little jobs inside of it. So, it was good learning about how much is in such little jobs"<br>P4: "I found it quite engaging um because yeah I was like actually interested about it."<br>P7: "Mhm, I liked the card I got from the body image person. Yeah, I still have the card at home. It's on my table."<br>P7: "Yeah. I took one of the leaflets home for an undergraduate one. I should have took a graduate one too. But like I just took an undergraduate one. I showed my mum it and stuff. Erm, I colour in better and I have more patience when colouring. That's my new skill."<br>P7: "And I- I- I- take pictures a lot more so I don't have any storage left. I take a lot of pictures. And, it opened my eyes to new food."<br>P8: "The art was really nice because we were all sitting down talking still discussing um and just expressing ourselves through physical like actions as well as painting which was really relaxing as well."	P6: "Just from like I think it's useful just to talk about it you know cause it's not something people talk about much."<br>P8: "Like how different people deal with it, what goes through their mind. How so many different things can lead to having mental health issues or having mental health problems and how people deal with stuff like that. That really made me more, that kind of opened up my outlook to it and stuff"<br>P11: "I enjoyed you know learning all that stuff about our body and with other people as well and like wondering about their insecurities and why they're insecure sometimes."<br>P11: "Well I learned how to identify like when people feel uncomfortable or in certain situations that they don't want to be in."<br>P11: "also listen to others and respect their decisions."<br>P11: "Yeah, I think they should because then it'll just help them understand everyone and not just the people in their area and community, just everyone around them as well."<br>P12: "everyone can connect with it and everyone can find something that resonates with them within the workshop."	

P8: “Yeah we visited the Cuban exhibition. That was pretty interesting because I don’t know-there was a lot of things I didn’t see before as well as you see. It was a really big campus so we got to see a lot of things I didn’t think I would see through universities and then you're more open to the idea of it.” P10: “I did get to hear a bit about the-the different types of sciences and I got to share some fun facts which was you know fun um and going to the wet lab helped me see how I would further like go into biology and different sciences because I plan on going to med school so it’s just like a- a snippet of it.” P10: “I like studying the human body and the way it works so just going around there and seeing like different exhibitions and art projects from people was helpful in my own personal studies of the body and my work with sculpting.” P10: “Definitely, the museums and the exhibitions just from- just because I learned new things about bodies and also, I do- I like zoology as well and animals” P10: “I wanna go back to that human exhibition um I wanna- I wanna actually- I wanna bring my sketchpad with me as well so I can yeah just do a bit of drawing with it.” P10: There were so many books that I wanted to read but I couldn’t stop and read them it was rather annoying. P12: “it was very helpful for me to like understand a lot of film work and broadcasting.”	

P12: “I got to contribute a lot to it”<br>P12: “When I was writing, when I was erm doing the script for the movie we are creating with another youth club I kinda like remembered their voices in the back of my head with like certain points they made and yeah.”<br>P12: “Yeah the body image yeah it was a lot about body confidence and I think I was going through something at that time with my body image and I think that workshop was like a godsend like it really opened my eyes a lot”<br>P12: “I kept it in um, I use it as like a bookmark sometimes like I keep it in the middle of my book and then if I open the page I see it and it just like reminds me of the workshop and everything and when I’m feeling down sometimes I’ll look at it and I’ll realise that it’s not all bad.”<br>P12: “everyone can connect with it and everyone can find something that resonates with them within the workshop.”	

Table D2.
All quotations related to superordinate theme 2: positive mental health outcomes

Superordinate theme 2: Positive mental health outcomes	
Self-understanding	Emotional expression and regulation
P1: “It did that’s why I put my hand up at one point and I said aw like there’s some points where I’m like yeah like I am pretty girl but when I look at instagram and all of that then then I’m like aw but maybe I’m not so pretty like you get it so?”<br>P1: “Like other people see you in a way that aw like yeah you’re not like ugly or very beautiful girl blah blah blah so I think like it shouldn’t like think about- you shouldn’t think too badly or too negative about how you look and yeah”<br>P4: “Yeah, we were thinking how- because- it allowed me to think about other people’s point of view but also from my own so- but like in a positive way- so sometimes you’ll dwell on something which isn’t actually isn’t as a big as it is and you think if a random stranger was to see you on the street was it really the first thing they’d notice?”<br>P5: “Um I mean I learned that uh people stereotyping isn’t like good. It's like- even jokes like you can’t really make jokes of it cause it’s not funny at the end cause like it could be affecting somebody's life.”<br>P7: “I started to think about it more, even then it took even longer than I wanted it to but I still thought about it. Sometimes I do feel like people are watching me on my way home. But not like, erm, secret detective watching or something, and I know this has nothing to do with the…”<br>P7: “In the covid pandemic in the beginning I I erm I didn’t wanna stay at home I wanted to leave the house. I did not want to stay inside. I felt, I felt I was suffocating then when I when I had a chance to go outside, I was like I don’t wanna go outside. It’s peaceful. No one’s bothering me, I have no homework. I have no classwork. I do not attend google meet and if I do, I’m watching anime the whole entire time. One time I didn’t write anything in my history or geography book for a whole month. I felt so proud of myself.”	*Emotional expression*<br>P4: “we was able to like- kinda like go deeper.”<br>P9: “I would say, I would say like it’s life changing to the point like you have to express you have to talk like some people out here do not talk to nobody and with these workshops it makes you more confident to talk to other people, explain how you feel, yeah.”<br>P11: “Um well about talking about mental health um well I enjoy like talking about it with other people”<br>P12: “So I feel like these workshops helped me a lot with my emotions.”<br><br>*Emotional regulation*<br>P8: “I kind of liked the art. The art was really nice because we were all sitting down talking still discussing um and just expressing ourselves through physical like actions as well as painting which was really relaxing as well.”<br>P8: “It was quite calming to be honest”<br>P9: “Erm, this whole thing actually changed my mental health. Mainly because, like, mainly because like I had like something, I don’t know how to say it like but like it distracted me from what I like am heading and I like when stuff distracts me because I’m mostly thinking about like what I’m doing and how I look blah blah blah but this didn’t. It made me express how I feel kind of thing.”<br>P12: “Yeah, I feel like one of the workshops it was talking about how to combat negative emotions. I feel like that helped me a lot”<br>P12: “the workshop can still help all of us with our individual emotions.”<br>P12: “So I feel like these workshops helped me a lot with my emotions.”

P8: “you can learn things about yourself in a way”
P8: “you kinda learn things about yourself in a way”
P9: “I kind of didn’t like it but at the same time I had to like get it out one way or another. But like yeah, I don’t know. I don’t like telling people my business. I keep everything confidential. With me like telling other people how like I feel and all of that shit um-“
P9: “Erm, this whole thing actually changed my mental health. Mainly because, like, mainly because like I had like something, I don’t know how to say it like but like it distracted me from what I like am heading and I like when stuff distracts me because I’m mostly thinking about like what I’m doing and how I look blah blah blah but this didn’t. It made me express how I feel kind of thing.”
P10: “it changed my perception on the social identity and stereotypes”
P10: “so that like opened my eyes to it because I hadn’t really had to think about it”
P12: “I think I was going through something at that time with my body image and I think that workshop was like a godsend like it really opened my eyes a lot”
P12: “It helps me a lot with realising that like I’m not perfect, I can’t be perfect, there’s no point of striving to be perfect. All I can do is trying to be the best I can be, but I know that’s not going to be perfect and that doesn’t have to be perfect for me to be good enough.”
P12: “Yeah, I feel like one of the workshops it was talking about how to combat negative emotions. I feel like that helped me a lot because I never really had like a sort of system or like coping mechanisms to deal with the negativity. I just sort of pushed it down and let it all bottle up and then I explode and then I realised that that’s not healthy for me and yeah.”
P12: “I feel like the body image one helped me a lot with um understanding my emotions and connecting with them and realising I’m a very emotional person and I then um I think it helped me realise that it’s ok to be emotional it’s just how you handle it that matters.”
P12: “yeah I feel like it helped me realise that through Covid I became a better person. Like through lockdown it gave me,

even though we were for so long trapped in our houses, it gave me time to myself to actually realise what was going on because before I would just bottle it up and I would go out and I would just forget about it and say yeah I'll figure this out later duh duh duh duh duh. But when I was locked at home all I could do was think about it and I realised there was no point thinking about it I'm not gonna do anything to change it."
P12: "and it focuses on… how you view yourself and yeah."

Change in negative perceptions
P1: "Like other people see you in a way that aw like yeah you're not like ugly or very beautiful girl blah blah blah so I think like it shouldn't like think about- you shouldn't think too badly or too negative about how you look and yeah"

P4: "so sometimes you'll dwell on something which isn't actually isn't as a big as it is and you think if a random stranger was to see you on the street was it really the first thing they'd notice?"

P5: "Um I mean I learned that uh people stereotyping isn't like good. It's like- even jokes like you can't really make jokes of it cause it's not funny at the end cause like it could be affecting somebody's life."

P9: "Erm, this whole thing actually changed my mental health. Mainly because, like, mainly because like I had like something, I don't know how to say it like but like it distracted me from what I like am heading and I like when stuff distracts me because I'm mostly thinking about like what I'm doing and how I look blah blah blah but this didn't. It made me express how I feel kind of thing."

P10: "Not really career paths but it changed my perception on the social identity and stereotypes um when it was on the same day as the human exhibition but there was the guy speaking-"

P12: "It helps me a lot with realising that like I'm not perfect, I can't be perfect, there's no point of striving to be perfect. All I can do is trying to be the best I can be but I know that's not going to be perfect and that

doesn't have to be perfect for me to be good enough."

Table D3. All quotations related to superordinate theme 3: aspects of the workshops which impeded positive mental health outcomes

Aspects of the workshops which impeded positive mental health outcomes		
Lack of engagement	Negative perception	Lack of topic relevance
P7: "The other ones were not as fun."<br>P7: "No offence to the people doing the BBC one but it was absolutely boring. I left halfway through and then I had to come back coz I was forced back but like it was really boring, and they were telling like all about themselves and I know it was rude to leave but I was so bored don't blame me. And the art one I got sharpie on my favourite jeans. I got sharpie on my favourite jeans. Sharpie leaked."<br>P7: "Would you recommend…? …Two, no unless you wanna sit down and listen to a lecture."<br>P9: "Like, I don't know. I like art but it wasn't like, it wasn't, it was just dry."<br>P12: "I know some other people that are not as patient like they would get sort of agitated."<br>P12: "I remember there was like other people like I just wanna go home"<br>P12: "I feel a lot of people can feel like they are just in another PSHE lesson or something like that."	P2: "to be honest with you I didn't really- I just thought it was going to be like a boring day at school"<br>P8: "cause at first it can be a thing like, ah I don't wanna do this I don't wanna do that<br>P8: I was like hm do I really wanna do this"<br>P12: "I thought it was gonna be like oh my god they're just gonna ask questions about the pandemic"<br>P12: "because I feel like most people when they see it for the first time they're bored or they're only in it for like the vouchers"	P6: "more for other people yeah probably. Like for me do I have mental health? I don't know."<br>P6: "No cause I don't- I don't care to be honest."<br>P6: "My mum calls- like my mum would call me fine and then my sister would call me like skinny but then they all would call me fat so like for them I'm everything. For me I just don't really care. Like who cares?"<br>P6: "I'm clearly not that fat that Thorpe Park says you can't go on the rides so I'm calm."<br>P7: "Well, I don't think about my mental health. I actually try and stay as far away from it as possible."

Topic	Item No.	Guide Questions/Description	Reported on Page No.
		correction?	
**Domain 3: analysis and findings**			
Data analysis			
Number of data coders	24	How many data coders coded the data?	9
Description of the coding tree	25	Did authors provide a description of the coding tree?	9
Derivation of themes	26	Were themes identified in advance or derived from the data?	9
Software	27	What software, if applicable, was used to manage the data?	8
Participant checking	28	Did participants provide feedback on the findings?	9
Reporting			
Quotations presented	29	Were participant quotations presented to illustrate the themes/findings? Was each quotation identified? e.g. participant number	10
Data and findings consistent	30	Was there consistency between the data presented and the findings?	16-19
Clarity of major themes	31	Were major themes clearly presented in the findings?	16-19
Clarity of minor themes	32	Is there a description of diverse cases or discussion of minor themes?	16-19

Developed from: Tong A, Sainsbury P, Craig J. Consolidated criteria for reporting qualitative research (COREQ): a 32-item checklist for interviews and focus groups. *International Journal for Quality in Health Care*. 2007. Volume 19, Number 6: pp. 349 – 357

Once you have completed this checklist, please save a copy and upload it as part of your submission. DO NOT include this checklist as part of the main manuscript document. It must be uploaded as a separate file.
