# Supplementary material for: Impact of co-created mental health and life-skill workshops with 12-to-16-year-olds from black and mixed ethnic groups during COVID-19 in the UK: a qualitative study
Source: BMJ Open. 2026 May 26;16(5):e107310. doi: 10.1136/bmjopen-2025-107310 (PMC13218165; doi:10.1136/bmjopen-2025-107310)
Supplement: Supplementary file 2 [file bmjopen-2025-107310.reviewer_comments.pdf]

PEER REVIEW HISTORY

BMJ Open publishes all reviews undertaken for accepted manuscripts. Reviewers are asked to complete a checklist review form and are provided with free text boxes to elaborate on their assessment. These free text comments are reproduced below.

ARTICLE DETAILS

Title (Provisional)

The impact of co-created mental health and life-skill workshops with 12–to-16-year-olds from black and mixed ethnic groups during Covid-19 in the UK: A qualitative study

Authors

Pomfret, Isabelle; Wong, Keri Ka-Yee

VERSION 1 - REVIEW

**Reviewer**	**1**
**Name**	**Lea, Alexandra N**
**Affiliation**	**Kaiser Permanente, Division of Research**
**Date**	**22-Dec-2025**
**COI**	**None**

This qualitative study aimed to characterize explore the impact, benefits, and challenges of co-created mental health psychoeducational workshops for Black and Asian mixed ethnic group (BAME) adolescents in London. The manuscript presents clear themes and rich participant quotations. The writing is generally coherent and well organized, but an additional grammatical review is needed. For example, there are many missing commas and acronyms should be written as the full name followed by the acronym in parentheses, not vice versa.

Some additional feedback to consider:

In the introduction, are you saying that mental health symptoms rose by those amounts or just comparing those amounts to what was reported by white counterparts? The sentence on lines 48-53 is not clear.

Additional Information about the number and types of workshops offered would be useful to contextualize findings. Additional details about how the study sample reflects the overall workshop attendance would also strengthen the paper.

Additionally, this study says that it is focused on how these workshops were received by BAME youth during COVID but the discussion does not really mention the pandemic and

how that experience may have impacted the experiences shared by participants at all. For example, were these workshops virtual or in person? Were they the only means these adolescents had for social interaction? The role that the pandemic had in influencing these results is not discussed.

If the author is going to list strong relationships between the student researchers conducting the interview and the participants as a major strength, please add additional details about this in the methods section. For example, were the interviewers the same individuals who led the workshops over the course of the year? As it is written, there is no justification for that statement in the manuscript.

**Reviewer**	**2**
**Name**	**Somprasert, Chomchuen**
**Affiliation**	**Mahidol University, MU Alumni**
**Date**	**12-Jan-2026**
**COI**	**Mental health and wellness**

681222. BMJ

Overall

1.The conclusion could be summarized the theme form content analysis and more focus about the specific coping strategies that effective to deal with the current health problems (Covid -19).

2.On page 9 : The triangulation method should be used to validate the data .

3.On page 10 line # 27 (10 participants-no described) When the researcher explained the results from conten anlysis that mean the theme /subtheme could be resulted from the frequency of code then narrative writing and not back to the number of participants.

4 And the same reason as # 3 : On page 10 line# 46 (9 /no desscribed)/

On page 11 line# 13(3 /no desscribed)

On page 12 line# 28(8 /no desscribed) # 49(4/ no described)

On page 14 line# 3 (3 /no desscribed) # 26(4/ no described)

5. Table 1-2-3 By QL presentation the data and content analysis should be narrative writing and presented the topic of theme /sub theme and intext reference by the Illustrative quotes that induction from code to theme/ subtheme. The table

showed be showed the digital data and other such as percentage Mean ,Standard deviation etc. Please concised overall.

6 Discussion should be followed through the theme and subtheme.

VERSION 1 - AUTHOR RESPONSE

Reviewer 1 (R1):
This qualitative study aimed to characterize explore the impact, benefits, and challenges of co-created mental health psychoeducational workshops for Black and Asian mixed ethnic group (BAME) adolescents in London. The manuscript presents clear themes and rich participant quotations. The writing is generally coherent and well organized, but an additional grammatical review is needed. For example, there are many missing commas and acronyms should be written as the full name followed by the acronym in parentheses, not vice versa.

- **Response**: Thanks for raising this. We have now conducted a thorough proofread of the manuscript to correct these grammar errors.

R1: In the introduction, are you saying that mental health symptoms rose by those amounts or just comparing those amounts to what was reported by white counterparts? The sentence on lines 48-53 is not clear.

- **Response:** Thank you. We have now rephrased the sentence in lines 48-53, now lines 162-163, for clarity:
- "BAME young people reported increased levels of anxiety (11.4%), suicidal thoughts (26.6%), and self-harm (29.5%), compared to their White peers at 3%, 18.1%, and 24.9%, respectively" .

R1: Additional Information about the number and types of workshops offered would be useful to contextualize findings. Additional details about how the study sample reflects the overall workshop attendance would also strengthen the paper.

- **Response:** Thank you. We have included the following changes in lines 228-239 to clarify the number and types of workshops offered.
- "A total of five in-person workshops were co-created and delivered with and for young people directly to address the impact of the Covid-19 pandemic on their lives. Workshop 1 involved participants taking a campus tour as an introduction to the university environment. Workshop 2 involved a journalist who spoke about the power of stories and the interpretation of our own story and experiences before sharing their career journey. Workshop 3 focused on the topic of body image and the impact of social media, featuring a talk from a clinical psychologist on coping skills. Workshop 4 involved social psychologists who led conversations on in-group/out-group identity, exploring topics such as discrimination, prejudice and stereotypes. Workshop 5 concluded with a creative session, where young people and members of the research team co-produced a single piece of artwork reflecting on what they had learned throughout the programme." […] "Workshop attendance ranged from 8 to 15 participants, with around 20 young people regularly attending this youth club."

R1: Additionally, this study says that it is focused on how these workshops were received by BAME youth during COVID but the discussion does not really mention the pandemic and

how that experience may have impacted the experiences shared by participants at all. For example, were these workshops virtual or in person? Were they the only means these adolescents had for social interaction? The role that the pandemic had in influencing these results is not discussed.

- **Response:** Thanks for raising this point. We have reworked the manuscript to reflect the role of the pandemic. To clarify, the workshops were in person and directly responded to each of the 'impact areas' that young people had voiced as being a result of the Covid-19 pandemic. The workshops took place throughout 2022, post- lockdown easing and at a time when some people were still readjusting to the return of social interactions and events. The workshops represented a welcome return to social interaction. We have now clarified this in the Discussion section on how the broader pandemic context may have shaped young people's experiences, including their appreciation of in-person connection and group-based activities, and how this context may have influenced the findings.
- An example of this addition is lines 403-405 in the manuscript reading:
- "Crucially, the workshops were delivered in person shortly following the easing of Covid-19 restrictions, and the sessions represented a meaningful return to face-to-face group activities."

R1: If the author is going to list strong relationships between the student researchers conducting the interview and the participants as a major strength, please add additional details about this in the methods section. For example, were the interviewers the same individuals who led the workshops over the course of the year? As it is written, there is no justification for that statement in the manuscript.

- **Response:** Thanks for the suggestion. Yes, the interviewers were the same individuals who recruited, listened, co-produced and led the workshops with and for young people throughout the project. The manuscript has now been updated to reflect this information in lines 248-250.
- "The same group of student researchers were consistently present at the youth centre, in workshop sessions and in conducting the focus groups and interviews, strengthening their ties with the young participants with each interaction."

Reviewer 2 (R2): Overall the conclusion should be summarized the theme form content analysis and more focus about the specific coping strategies that effective to deal with the current health problems (Covid-19)

- **Response:** Thanks for your suggestion. We have rephrased our conclusion paragraph to more explicitly detail how each theme, addressed by each workshop, promotes a specific coping strategy for young people living with current inequalities who have experienced the COVID-19 pandemic. This section between lines 513-523 now reads:
- "This semi-structured interview study investigated young people from minority ethnic communities' experiences of co-created mental health and life-skill workshops and their impact as they continue to recover from the COVID-19 pandemic. The themes highlighted how the workshops imparted crucial wellbeing skills, such as emotional regulation, self-acceptance and long-term coping strategies, alongside social benefits, fostering a truly supportive community among their participants. Collectively, these elements supported participants' capacity to navigate ongoing challenges during post-pandemic recovery. Future

workshops should prioritise participant enjoyment, engagement, community building, and the coping skills gained from attending, and continue to tailor community support for young people. This workshop feedback is but a start to a critical area of strategic community preventative intervention for mental well-being."

R2: On page 9 : The triangulation method should be used to validate the data .

- **Response:** Thanks for your comment. Whilst we acknowledge that we did not triangulate qualitative theories to examine young people's lived experience of the COVID-19 pandemic - we did verify and validate the codes, reading, and interpretation of the transcripts as a team of three researchers (I.P., Z.B., & K.W.). Moreover, the project also collected questionnaire data assessing mental health outcomes that could be used for triangulation but is beyond the scope of the current paper and is thus not presented here.

R2: On page 10 line #27 (10 participants-no described) When the researcher explained the results from content analysis that mean the theme/subtheme could be resulted from the frequency of code then narrative writing and not back to the number of participants.

- **Response:** Thanks for your comment. We believe you are asking us to include more description in this section? If so, thanks for the suggestion. We have now included the following amendment on page 10 line #27, now line 303 of the text to read:
- "Nearly all participants (n=10) noted that the workshops were enjoyable in some way and described meaningful engagement with activities that aligned with their interests."
- We have written "nearly all" as a narrative descriptor alongside the participant count "(n=10)".

R2: And the same reason as #3: On page 10 line #46 (9/no described)/
On page 11 line #13 (3/no described)
On page 12 line #28 (8/no described) #49(4/no described)
On page 14 line #3 (3/no described) #26(4/no described)

- **Response:** We appreciate this important methodological point. In response, we have revised the Results section to add in narrative descriptions without solely relying on counts of participants as evidence. Narrative phrasing (e.g., "many," "several") has been used alongside participant counts to better reflect the interpretive nature of the analysis.

R2: Table 1-2-3. By QL presentation, the data and content analysis should be narrative writing and present the topic of theme/sub-theme and in-text reference by the Illustrative quotes that induce from code to theme/sub-theme. The table showed be showed the digital data and other such as percentage Mean, standard deviation etc. Please concised overall.

- **Response:** Apologies but we are struggling to understand what your comment means especially what "QL" stands for. Please can you clarify this? We have inferred that more narrative writing is needed and have added in contextual references to the illustrated quotes.
- For example, lines 331-335 read "Therefore, such initiatives have the potential to support the development of long-term emotional resilience and act as tools to manage future tumultuous life events." – adding in more description following the quotation.

R2: Discussion should be followed through the theme and subtheme.

- **Response:** Thanks for your comment. We have now revised our Discussion section to centre more on the themes and subthemes generated from our project. This is reflected in the revised sections in lines 400-478.
- As an example, lines 451-455 were added. “The workshops appeared to provide not only information but also emotional validation, enabling participants to reframe negative self-perceptions and feel less alone in their experiences. For some participants, this process of reflection and discussion facilitated a shift towards greater self-compassion and confidence.”

Many thanks for providing useful feedback that we have used to enhance our manuscript. This is the end of our response to reviewer comments.

VERSION 2 - REVIEW

**Reviewer**	**1**
**Name**	**Lea, Alexandra N**
**Affiliation**	**Kaiser Permanente, Division of Research**
**Date**	**24-Apr-2026**
**COI**	

The author has been responsive to reviewer comments and the manuscript has been substantially improved.
